# Worst-case scenario analysis of physical demands in elite men handball players by playing position through big data analytics

**DOI:** 10.5114/biolsport.2023.126665

**Published:** 2023-09-27

**Authors:** Antonio Carton-Llorente, Demetrio Lozano, Virgilio Gilart Iglesias, Diego Marcos Jorquera, Carmen Manchado

**Affiliations:** 1 Universidad San Jorge, Autov A23 km 299, 50830 Villanueva de Gállego, (Zaragoza), Spain; 2 Department of Computer Science and Technology, Polytechnic School, University of Alicante, 03690 San Vicente del Raspeig, Spain; 3 Faculty of Education, University of Alicante, 03690 San Vicente del Raspeig, Spain

**Keywords:** Most demanding scenarios, Peak demands, Running pace, Official matches, LPS

## Abstract

The physical demands of intermittent sports require a preparation based, by definition, on high-intensity actions and variable recovery periods. Innovative local positioning systems make it possible to track players during matches and collect their distance, speed, and acceleration data. The purpose of this study was to describe the worst-case scenarios of high-performance handball players within 5-minute periods and per playing position. The sample was composed of 180 players (27 goalkeepers, 44 wings, 56 backs, 23 centre backs and 30 line players) belonging to the first eight highest ranked teams participating in the European Men’s Handball Championship held in January 2022. They were followed during the 28 matches they played through a local positioning system worn on their upper bodies. Total and high-speed distance covered (m), pace (m/min), player load (a.u.) and high-intensity accelerations and decelerations (n) were recorded for the twelve 5-min periods of each match. Data on full-time player average and peak demands were included in the analysis according to each playing position. A systematic three-phase analysis process was designed: 1) information capture of match activities and context through sensor networks, the LPS system, and WebScraping techniques; 2) information processing based on big data analytics; 3) extraction of results based on a descriptive analytics approach. The descriptive cross-sectional study of worst-case scenarios revealed an ~17% increment in total distance covered and pace, with a distinct ~51% spike in high-intensity actions. Significant differences between playing positions were found, with effect sizes ranging from moderate to very large (0.7–5.1). Line players, in particular, showed a lower running pace peak (~10 m/min) and wings ran longer distances at high speed (> 4.4 m/s) than the rest of the field players (~76 m). The worst-case scenario assessment of handball player locomotion demands will help handball coaches and physical trainers to design tasks that replicate these crucial match moments, thus improving performance based on a position-specific approach.

## INTRODUCTION

Handball is a dynamic team sport presenting physical, physiological, technical, and tactical demands as well as cognitive challenges [1]. During competitions, the game is divided into different phases (offensive and defensive). Players (six players and one goalkeeper) attempt to create space to shoot effectively, while the defence tries to prevent them from doing so. Such play leads to demanding physical confrontations between players followed by variable recovery periods [2].

Quantifying workload in intermittent sports poses a challenge for sport scientists. Indeed, they have to quantify not only the physiological demands of the movements but especially their mechanical demands. Handball players must be able to sprint, cut, jump, shoot, block and push [3, 4] across variable short-duration and maximum-intensity combinations. In this regard, previous research has focused on describing the conditional demands of intermittent sports by combining internal load (heart rate data) and external load variables (mainly through video analysis) as a prerequisite to tailoring the training sessions to the players’ actual demands. Notably, these studies have identified an effect of the contextual variables, such as playing position, on the physical demands of soccer [5], basketball [6] and handball [7–9], paving the way towards training individualisation in intermittent sports.

In recent decades, many companies have developed ultra-wideband systems to collect real-time data in outdoor sports. This has made it possible to better analyse players’ physical demands during training and competition using Global Positioning System (GPS) technology [8, 10, 11]. This novel technology has been adapted to indoor sports using local positioning systems (LPS). In particular, it has been implemented in handball by the European Handball Federation (EHF), Select and Kinexon, together with the Kinexon tracking system for handball players (Kinexon: München, Germany; Select Sport 1947: Glostrup, Denmark). Thanks to its recent validation [12, 13], it has been used in research on handball physical demands [4, 14–20] and other indoor team sports [21–24].

LPS studies have traditionally quantified average physical demands in different team sports during training and competition using variables such as distance run at different speeds (in m); time at different speeds (in s); and number (n) of high-intensity accelerations (HIA), decelerations (HID), and changes of direction [25, 26]. Of note, a number of companies recently developed a composite variable called “player load” in an attempt to capture and synthesise 3-axis accelerations, decelerations, impacts and changes of direction with a unique number, expressed in arbitrary units (a. u.). This number has been used to establish differences between playing positions [18]. Researchers have since stated, however, that due to the fluctuating nature of team sports, the average demands approach underestimates actual player workload during intermittent efforts. The reason advanced is that it does not sufficiently weight the effects of high-intensity actions concentrated in the most strenuous phases of an actual competition [27, 28]. As a result, worst case scenario (WCS) analysis in team sports has emerged. This novel approach aims to quantify the highest possible demands within brief time intervals, which are also known as the most demanding passages. WCS are defined as short time periods of maximum physical performance (distance covered at high running speed) throughout a match [29, 30]. The fixed duration method was the first attempt to quantify WCS [31] and consisted of dividing the match from start to end into fixed 5-minute periods. It aimed at reflecting more accurately the physiological and mechanical demands of intermittent sports [32, 33]. From this standpoint, identifying and quantifying the highest demands during a competition has become of great value to team sport coaches and physical trainers: the knowledge helps them to design training tasks that reproduce the most demanding contexts – not only average demands.

To date, team sport WCS analyses have been based on time windows ranging from 30 seconds to 10 minutes, relative demands being higher in shorter time windows [10, 23]. Only one study [23] has analysed WCS in handball however, and players from just one team were included in the study. A WCS analysis requires processing a large amount of information as well as heterogeneous data sources and formats. It is thus necessary to devise a system that homogenises and automates this process in order to obtain the information in a limited time and with adequate quality. As a result, we designed a modular and comprehensive system based on Big Data Analytics. It allowed the proposed methodology to be implemented with the objective of capturing, storing, processing, and analysing the information that was necessary for this study. Therefore, the main objective of the present study was to describe the WCS of maximal time-motion demands in elite male handball players during an official competition and to identify differences between playing positions.

## MATERIALS AND METHODS

### Experimental approach to the problem

**FIG. 1:**
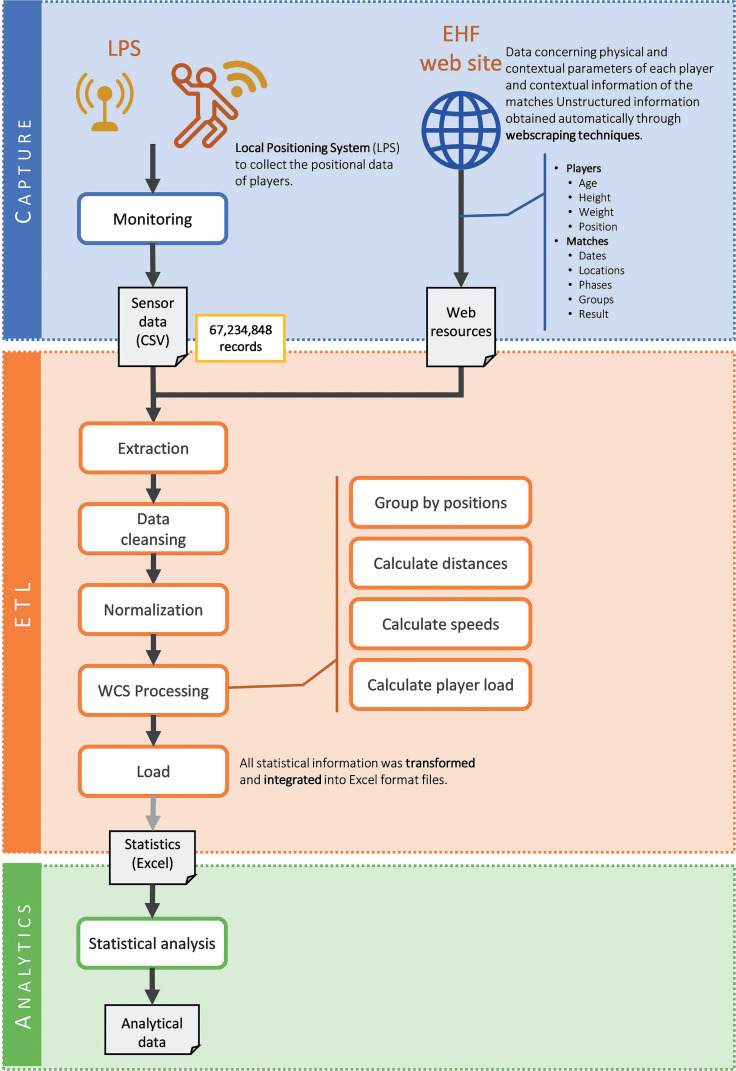
Methodology of the comprehensive handball system proposed.

A cross-sectional, observational study was implemented to describe time-motion WCSs in handball players and identify the differences between playing positions: goalkeepers (GK), wings (W), centre backs (CB), backs (B) and line players (LP). Results included the average values of 28 competitive official matches disputed in the European Men’s Handball Championship, held in Hungary and Slovakia in January 2022. In order to obtain and analyse the WCSs, a comprehensive system based on a sensors network, LPS and big data analytics was designed following the methodology described in Figure 1.

### Subjects

The data were obtained from the 180 players (27 GK, 44 W, 56 B, 23 CB and 30 LP) belonging to the top eight qualifiers of the European Handball Federation (EHF) Euro 2022. Anthropometric characteristics and the players’ age are presented in Table 1. All information (67,234,848 records) was collected from the official statistical data provided by the EHF and the routine monitoring of players during the competition (Figure 1, Capture layer). Ethics committee approval was therefore not required [34]. Nonetheless, the study followed the Declaration of Helsinki recommendations.

**TABLE 1:** Players’ physical characteristics (mean ± standard deviation).

Playing Position	n	Height (cm)	Body Mass (kg)	BMI (kg/m^2^)	Age (Years)
Centre back	23	190.8 ± 4.6	92.3 ± 6.8	25.3	28.8 ± 4.6
Left back	33	195.5 ± 4.3	97.1 ± 6.2	25.4	27.3 ± 4.3
Right back	23	192.7 ± 5.0	94.3 ± 5.5	25.4	28.2 ± 3.8
Line player	30	197.3 ± 4.9	105.1 ± 9.1	27.0	28.5 ± 4.5
Left wing	24	189.0 ± 4.9	88.2 ± 6.3	24.7	27.8 ± 3.2
Right wing	20	186.4 ± 4.4	83.6 ± 6.4	24.0	29.2 ± 5.4
Goalkeeper	27	195.0 ± 5.8	97.2 ± 8.7	25.6	30.4 ± 5.0
Total	180	192.9 ± 0.6	94.8 ± 9.5	25.5	28.5 ± 4.5

### Competitive match monitoring (Figure 1, Capture layer)

Each player’s 3D position data (x / y / z) were obtained in real time via a wearable inertial sensor (Kinexon SafeTag, Kinexon Precision Technologies, Munich, Germany) that was included in a top worn under the playing shirt, and that fit between the shoulder blades to avoid any inconvenience to the players. This tiny device (49 × 33 × 8 mm) weighs only 14 g and provides 9-axis inertial data (accelerometer, gyroscope, magnetometer) capable of recording accelerations/decelerations, rotation, and orientation angles (roll, pitch, yaw) with an update rate up to 60 Hz. The device has notably already been used for time-motion analysis in indoor team sports [20, 21, 35]. Indeed, it presents adequate between-device reliability (coefficient of variation around 5%) and a high level of agreement when compared to well-known systems such as GPS [12, 36].

The Kinexon system operates by means of triangulations between 9 antennae located around the handball court and connected to a server, and ten reference antennae acting as anchors. Setting, calibration and verification of the LPS system in all championship facilities followed the procedure described in the study by Manchado et al. [35].

The rest of the data necessary to perform the WCS analysis were collected from the EHF Website using WebScraping techniques.

### Data processing (Figure 1, ETL layer)

Total distance covered (m), high-speed running (HSR) (≥ 4.4 m/s) (m), running pace (m/min), player load (arbitrary units), HIA (> 2 m/s^-1^) (n) and HID (> -2 m/s^-1^) (n) were individually recorded for the twelve 5-min periods of each match, following official EHF timekeeping. The pace was calculated as the ratio between distance covered and playing time, and player load was expressed as the accumulated square root of the sum of the squared instantaneous rates of acceleration change in each one of the three planes [24, 37, 38].

The collected data were cleaned up and normalised before proceeding to the WCS processing phase. Thereafter, the 5-min period with the peak values for each player was selected for every variable.

Finally, in order to perform the subsequent data analysis, we conducted the loading process and all the information necessary for the study was transformed into Excel format files using an input format compatible with the statistical analysis tool.

Firmware and software versions employed in this study corresponded to the latest update of the aforementioned company (2019).

### Statistical analysis (Figure 1, Analytics layer)

Descriptive statistics are presented as means and standard deviations (SD) for all workload variables. Mean and peak demands were averaged for each fixed 5-min timespan and playing position. Playing time was recorded only when the players were on court and only full-time players (≥ 240 s for each 300 s time window) were included in the analyses.

The Kolmogorov-Smirnov test was conducted to confirm data distribution normality and Levene’s test for equality of variances. A separate one-way analysis of variance (ANOVA) was used to identify differences between playing positions, regarding both average and peak demands for the fixed 5-min time spans. Finally, Gabriel or Games-Howell post-hoc analyses were also conducted when appropriate to determine significant differences between playing positions. Effect sizes for all pairwise comparisons were also calculated using Cohen’s d, with 95% confidence intervals. Cohen’s d was interpreted as follows: trivial = 0 to 0.19, small = 0.2 to 0.59, moderate = 0.6 to 1.19, large = 1.2 to 1.99, very large = 2.0 to 3.99, and nearly perfect = 4.0 [39].

## RESULTS

**FIG. 2:**
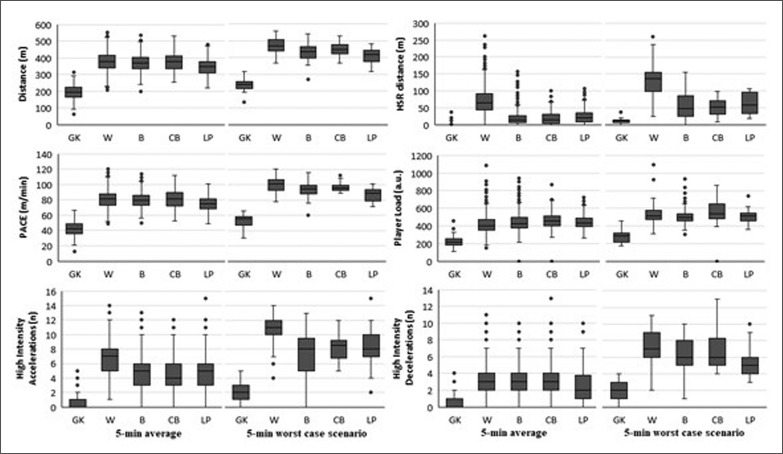
Performance analysis of 5-min time spans in average (left columns) and worst-case scenarios (right columns) according to the different playing positions, shown for full-time players.

Table 2 shows the WCS of the examined variables according to the different handball playing positions in fixed 5-min time spans. The one-way ANOVA showed significant differences between goalkeepers and all other positions (p < 0.001), with effect sizes ranging from very large to nearly perfect (2.0–5.1). After post-hoc testing, differences between line players and all other positions were found for peak running pace (effect sizes between 0.7 and 4.0). For their part, wing players presented longer distances covered at HSR (> 4.4 m/s) than the rest, except the centre backs (effect sizes between 1.7 and 3.2). Moreover, no differences were found between playing positions in terms of player load, excluding goalkeepers. The position-specific performance analyses in fixed 5-min windows, of both average and peak demands, are shown in Figure 2. Additionally, Table 3 reports the differences (in %) between average and peak demands for the aforementioned 5-min fixed time spans.

**TABLE 2:** Worst-case scenario analysis of 5-minute time spans during the 2022 European men’s handball championship. Effect size and statistically significant differences between playing positions in time-motion variables.

Variables	Wing (W)	Mean Difference	ES	Centre back (CB)	Mean Difference	ES	Back (B)	Mean Difference	ES	Line Player (LP)	Mean Difference	ES	Goalkeeper
Total distance (m)	469 ± 49	CB: 21 ± 13	0.5	447 ± 49	B: 12 ± 13 LP: 34 ± 14 GK: 212 ± 14*	0.3 0.8 5.1	435 ± 48	LP: 22 ± 11 GK: 199 ± 11*	0.5 4.3	413 ± 45	GK: 178 ± 13*	4.0	235 ± 42
		B: 34 ± 19*	0.7										
		LP: 55 ± 11*	1.2										
		GK: 233 ± 12*	4.9										

HSR distance (m)	133 ± 47	CB: 82 ± 10*	1.9	51 ± 26	B: -6 ± 10 LP: -11 ± 11 GK: 40 ± 11*	0.2 0.4 2.2	57 ± 41	LP: -5 ± 9 GK: 46 ± 9*	0.1 1.3	62 ± 29	GK: 51 ± 10*	2.3	11 ± 8
		B: 76 ± 8*	1.7										
		LP: 71 ± 9*	1.7										
		GK: 122 ± 9*	3.2										

Pace (m/min)	100 ± 10	CB: 2 ± 2	0.3	97 ± 9	B: -2 ± 3 LP: 10 ± 3* GK: 212 ± 14*	0.3 1.3 5.1	94 ± 10	LP: 7 ± 2* GK: 42 ± 2*	0.7 4.1	87 ± 8	GK: 35 ± 3*	4.0	52 ± 9
		B: 5 ± 2	0.7										
		LP: 12 ± 2**	1.2										
		GK: 47 ± 2**	4.9										

Player Load (a. u)	546 ± 139	CB: 9 ± 34	0.1	537 ± 177	B: 17 ± 38 LP: 21 ± 37 GK: 255 ± 30*	0.1 0.2 2	520 ± 119	LP: -4 ± 39 GK: 238 ± 39*	.04 2.2	516 ± 91	GK: 234 ± 34*	2.8	282 ± 73
		B: 26 ± 26	0.2										
		LP: 30 ± 30	0.2										
		GK: 264 ± 31*	2.2										

HIA (n)	10.6 ± 2.1	CB: 2.4 ± 0.6*	1.2	8.2 ± 2.0	B: 0.6 ± 0.6 LP: -0.2 ± 0.7 GK: 5.9 ± 0.5*	0.2 0.1 3.5	7.6 ± 2.7	LP: -0.8 ± 0.6 GK: 5.3 ± 0.5*	0.3 2.3	8.4 ± 2.6	GK: 6.1 ± 0.6*	2.9	2.2 ± 1.4
		B: 3.0 ± 0.5*	1.2										
		LP: 2.2 ± 0.6*	0.9										
		GK: 8.4 ± 0.4*	4.4										

HID (n)	6.8 ± 2.2	CB: .004 ± 0.6	0.0	6.8 ± 2.4	B: 0.8 ± 0.6 LP: 1.4 ± 0.6 GK: 4.7 ± 0.6*	0.4 0.7 2.6	6.0 ± 2.1	LP: 0.6 ± 0.5 GK: 3.9 ± 0.4*	0.3 2.1	5.4 ± 1.9	GK: 3.2 ± 0.4*	2.0	2.2 ± 1.1
		B: 0.8 ± 0.4	0.3										
		LP: 1.4 ± 0.5*	0.7										
		GK: 4.7 ± 0.5*	2.4										

ES: effect size; HSR: High Speed Running (> 4.4 m/s); HIA: High Intensity Accelerations (> 2m · s^-1^); HID: High Intensity decelerations (> 2m · s^-1^); Player Load (a. u.): sum of the squared rates of change in acceleration (also known as jerk) in each of the three vectors expressed in arbitrary units.

* Significant differences (p < 0.05); moderate = 0.6 to 1.19, large = 1.2 to 1.99, very large = 2.0 to 3.99, and nearly perfect = 4.0

**TABLE 3:** Performance variables of elite men handball players in fixed 5-min time spans: Position-specific Comparison between average and peak values.

Position Variables	5-min Average	5-min Worst Case Scenario	Difference (%)
**Goalkeeper**			
Total distance (m)	194 ± 39	235 ± 42	17
HSR distance (m)	1 ± 4	11 ± 8	91
Pace (m/min)	42.5 ± 9	52.3 ± 9	19
Player Load (a. u)	217 ± 50	282 ± 73	23
HIA (n)	0.8 ± 1.0	2.2 ± 1.4	65
HID (n)	0.6 ± 0.8	2.2 ± 1.1	72

**Wing**			
Total distance (m)	378 ± 56	469 ± 49	19
HSR distance (m)	70 ± 38	133 ± 47	47
Pace (m/min)	81 ± 11	100 ± 10	19
Player Load (a. u)	419 ± 118	546 ± 139	23
HIA (n)	6.7 ± 2.3	10.6 ± 2.1	37
HID (n)	3.3 ± 1.9	6.8 ± 2.2	52

**Back**			
Total distance (m)	369 ± 50	435 ± 48	15
HSR distance (m)	20 ± 23	57 ± 41	65
Pace (m/min)	80 ± 10	94 ± 10	15
Player Load (a. u)	448 ± 114	520 ± 119	14
HIA (n)	4.7 ± 2.2	7.6 ± 2.7	38
HID (n)	3.1 ± 1.9	6.0 ± 2.1	49

**Centre back**			
Total distance (m)	374 ± 54	447 ± 39	16
HSR distance (m)	19 ± 20	51 ± 26	63
Pace (m/min)	81 ± 11	97 ± 9	17
Player Load (a. u)	456 ± 108	537 ± 177	15
HIA (n)	4.5 ± 2.5	8.2 ± 2.0	45
HID (n)	3.2 ± 2.2	6.8 ± 2.2	53

**Line player**			
Total distance (m)	346 ± 49	413 ± 45	16
HSR distance (m)	24 ± 23	62 ± 29	61
Pace (m/min)	75 ± 9	87 ± 8	14
Player Load (a. u)	442 ± 79	516 ± 91	14
HIA (n)	4.9 ± 2.4	8.4 ± 2.6	42
HID (n)	2.5 ± 1.8	5.4 ± 1.9	54

HSR: High Speed Running (> 4.4 m/s); HIA: High Intensity Accelerations (> 2m · s^-1^); HID: High Intensity decelerations (> 2m · s^-1^); Player Load (a. u.): sum of the squared rates of change in acceleration (also known as jerk) in each of the three vectors expressed in arbitrary units.

## DISCUSSION

The aim of the current study was to profile the position-specific worst-case scenarios of locomotor demands in elite male handball players across fixed, 5-min time spans, and to identify differences between playing positions. The main findings were twofold: i) significant differences between playing positions were found in WCS locomotor demands with effect sizes ranging from moderate to very large (0.7–5.1) – wings performing longer distances at HSR (~55%) and line players showing the lowest pace among field players (~11%); and ii), 5-min WCS were revealed to be ~17% higher than the average demands in terms of total distance covered and pace, with a particular increase in high-intensity actions (~51%).

### Worst-case scenario differences between playing positions

Regarding WCS analyses, only two recent studies [23, 40] have described time-motion variables in top-level handball players. As in the current study, Fleureau [40] found that wing players endured the highest peak locomotor intensity (i.e., running pace) during matches (wings = 102 ± 7; backs = 95 ± 9; line players = 89 m/min). These results confirm our findings (100 ± 10; 95 ± 10; 87 ± 8 m/min, respectively), as line players also presented the lowest locomotion demands among field players – though their study revealed slightly lower effect sizes overall.

Nevertheless, HSR distance was significantly higher than in our study. It was not possible, however, to assess differences because of the thresholds defined in this study to classify HSR (> 4 m/s vs > 4.4 m/s). In addition, the present study analysed fixed 5-min intervals, while Fleureau’s study applied rolling averages to identify the WCS. Previous comparisons between these methods have shown that the rolling average approach identified 10% to 25% higher peak demands when using 5-min time windows [41, 42]. Therefore, peak demand may have been underestimated in the present study.

It is also worth mentioning that Fleureau [40] assessed only 11 players from one top French professional league team, whereas the current study monitored 180 players from 8 different national teams during 29 competitive matches, providing further robustness to the results.

On the other hand, the study by Garcia [23] compared overall locomotion demands of different team sports from the same club. In the case of handball, a generic field player showed values similar to those of the present study for the total distance run (< 6% of difference) but larger differences when comparing high-intensity actions (~50%). Again, the thresholds defined to classify HSR differed slightly from those used in the Kinexon system, which prevented us from assessing any agreement regarding high-intensity actions between both studies.

### Average vs peak demands

Average values have traditionally been used to profile the physical demands of intermittent sports. It has recently been suggested, however, that this latter approach may overlook the most demanding match-play phases, underestimating the physiological and mechanical effects of repeated high-intensity actions – which are so characteristic of these sports [28, 43]. Nevertheless, the evidence regarding WCS in handball remains scarce. We thus decided to include a descriptive analysis of the average demands in fixed 5-min periods to be able to compare our results with previous evidence, as well as to outline the percentage of change between the average values and the WCS.

Our results identified differences of ~17% in total distance covered and pace, and ~50% in high-intensity actions when comparing average and peak values during 5-min time spans. In this regard, previous research [40] suggested reductions of ~15% and ~37%, respectively, when compared 5- to 15-min periods.

Regarding average demands, a range of previous studies [4, 14–18, 20, 25, 26, 44] have quantified the position-specific handball physical requirements in different contexts (amateur/professional, male/female), with controversial results. Despite the fact that they described lower average running paces and total distances covered in their whole match analyses [4, 9, 18], peak values in Karcher’s and Luteberget’s studies resembled ours. Moreover, Karcher and Buchheit’s study [4, 18] also identified wing players as having the longest distances and high-speed actions (ES = 1.1) and pivots as having the least (ES = 1.7). Of note, these studies described a wider range of high-intensity actions (e.g., contacts, duels, jumps) using a notation system that hinders a comparison with LPS data [4].

Comparable results were found by Manchado [35] regarding average running pace and HSR distance according to playing position. Centre and left backs, however, were found to be the most demanding positions overall. In this regard, the works of Kniubaite [17] and Font [16] introduced the player load as an accurate metric to control external load during competition, describing centre backs as having the highest workload among field players. Conversely, our results confirmed no differences between playing positions, whether during average or peak demands. A possible explanation could be found in the studies of Povoas [9] and Karcher [4]: the characteristic actions of line players and backs include a greater number of duels, impacts and changes of direction than in the case of wingers. They may thus present similar or greater values in overall external load than the latter, but with lower locomotor demands.

Despite the reported findings, the current study presented some limitations. First, only full-time players were included in the analysis, preventing us from knowing the time-motion characteristics of attacking or defensive specialists. Additionally, the use of fixed time spans may lead to underestimating the most demanding phases during games compared to rolling averages. In this sense, a study profiling the different locomotor demands of attack and defence phases based on shorter time periods would allow the current knowledge gap to be filled. Moreover, a single, unified criterion is needed to determine locomotion categories in LPS analysis, as this lack of common criterion is currently preventing comparisons between studies. In this regard, future research should focus on handball characteristics based on a holistic perspective and include collisions, fights and impacts as well as peak locomotor activities in the game analysis, thus mirroring similar rugby studies [45, 46].

Despite these limitations, the present study on handball locomotion worst-case scenarios provides meaningful information making it possible to customise training drills to each player position and to better prepare players for peak demands during competitions.

## CONCLUSIONS

The position-specific description of handball locomotor peak demands showed that wing players cover longer total and HSR, whereas line players cover the shortest distances among all playing positions. These results have direct implications for the design of position-specific conditioning drills (e.g., short or long intervals, repeated sprints, etc.): practitioners should consider including more duels and impacts, and less running distance for line players, together with supplementary repeated sprint work for wingers. In addition, the traditional average method was found to underestimate worst-case demands over 5-min fixed periods by up to 51%. Therefore, when designing training sessions and rehabilitation protocols, coaches must be aware of the possible worst-case scenario, as the average-based approach may fail to prepare players for what they will actually experience during elite handball competitions. The performance of the present study required the design of an integral and modular system based on a sensors network, LPS, and big data analytics.

### Conflict of interest

The authors declared no conflict of interests.
